# Variation in multimorbidity prevalence by number of long-term conditions counted: primary care population cross-sectional study.

**DOI:** 10.23889/ijpds.v7i3.2053

**Published:** 2022-08-25

**Authors:** Clare MacRae, Stewart Mercer, Bruce Guthrie

**Affiliations:** 1 The University of Edinburgh

## Objectives

Multimorbidity poses a major challenge to healthcare systems worldwide, but there is no standardised approach to measuring multimorbidity. Existing studies include a median of 17 (IQR 11-23, range 2-285) long-term conditions (LTCs), making it difficult to compare or replicate research, particularly research examining the prevalence of multimorbidity.

## Approach

In this cross-sectional study of 1,168,620 people from the Clinical Practice Research Datalink, we extracted data on 99 LTCs defined by validated code-sets, including all conditions recommended as core by a recent Delphi study. We examined how the prevalence of multimorbidity defined as ≥2 LTCs, varied when including different numbers of LTCs in the measure. We calculated multimorbidity prevalence when including only the two most prevalent LTCs, repeating the calculation for 3,4,5, through to 99 LTCs, added in order of descending prevalence. Results were stratified by age, sex, and socioeconomic status (SES).

## Results

Using only the two most prevalent LTCs, 16.8% of people had multimorbidity, compared to 39.3% for 10 LTCs, 42.0% for 25 LTCs, 42.5% for 50 LTCs, 42.6% for 75 LTCs, and 42.6% for all 99 LTCs. Whole population prevalence was essentially stable once 46+ LTCs were included (estimated prevalence change from adding any additional LTC <0.01%) and this was true in all age strata. The absolute difference in estimated prevalence using 99 versus using two LTCs was smallest in younger people (0.3% difference aged 0-4yrs), large in older people (20.4% ≥90yrs), largest in middle age (37.0% 55-59yrs), and was socially patterned, being lowest for people living in the most affluent areas (SES decile 1= 24.2%) and highest in the most deprived areas (SES decile 10=28.9%).

## Conclusion

Estimates of multimorbidity prevalence vary markedly depending on how many LTCs are counted, with absolute differences in estimated prevalence largest for people in middle-age and with lowest SES. Further research is needed to support implementation of a uniform approach to defining how many conditions to include in multimorbidity measures.

